# Expression of pluripotency-related genes in human glioblastoma

**DOI:** 10.1093/noajnl/vdab163

**Published:** 2021-12-20

**Authors:** Álvaro Fabrício Lopes Rios, Daniela Pretti da Cunha Tirapelli, Mucio Luiz de Assis Cirino, Andressa Romualdo Rodrigues, Ester S Ramos, Carlos Gilberto Carlotti

**Affiliations:** 1 Laboratory of Biotechnology, Center for Biosciences and Biotechnology, North Fluminense State University, Campos dos Goytacazes, Rio de Janeiro, Brazil; 2 Department of Surgery and Anatomy, Ribeirão Preto Faculty of Medicine, University of São Paulo, Ribeirão Preto, São Paulo, Brazil; 3 Laboratory of Morphofunctional and Integrated Practices, Franca Medical School, University of Franca, Franca, São Paulo, Brazil; 4 Department of Genetics, Ribeirão Preto Faculty of Medicine, University of São Paulo, Ribeirão Preto, São Paulo, Brazil

**Keywords:** cancer, gene expression, pluripotency, stem cell

## Abstract

**Background:**

Cancer is a group of heterogeneous diseases characterized by several disruptions of the genetic and epigenetic components of cell biology. Some types of cancer have been shown to be constituted by a mosaic of cells with variable differentiation states, with more aggressive tumors being more undifferentiated. In most cases, undifferentiated tumor cells express associated embryonic markers such as the OCT4, NANOG, SOX2, and CARM1 genes. The ectopic or reminiscent expression of some master regulator genes of pluripotency has been indicated as the cause of the poorly differentiated state of tumors, and based on the evidence of some reports, can be used as a possible therapeutic target. Considering this information, a more detailed investigation of the expression of pluripotency-associated genes is necessary to evaluate the roles of these genes in the etiology of some tumors and their use targets of therapy.

**Methods:**

The expression of four pluripotency-related genes was investigated (OCT4, NANOG, SOX2, and CARM1) in the most malignant primary human brain tumor, glioblastoma (GBM).

**Results and Conclusion:**

The results demonstrated a signature of OCT4/SOX2/CARM1 genes and a significant increase of CARM1 expression in GBM cases.

**Key Points**

- Study of human glioblastoma samples.
- Identification of a signature of pluripotency-related genes that may help to elucidate tumorigenesis and treatment of GBM.

**Importance of the Study**

This study describes some characteristics of glioblastoma, a tumor type is highly aggressive usually fatal. Identifying a pluripotency-related gene signature is a very important step for medical science.

Glioblastoma (GBM) or astrocytoma grade IV according to the World Health Organization (WHO) classification is the most aggressive and common of all primary brain tumors, accounting for approximately 40% of all primary and 78% of all malignant central nervous system tumors.^1,2^ Mutations (TP53, p16INK4a, p14ARF, and PTEN), amplifications (EGFR and CDK4/6) and loss of heterozygosity (LOH) of several chromosomes (1p, 6q, 9p, 10p, 10q, 13q, 14q, 15q, 17p, 18q, 19q, 22q, and Y) are among the major genetic alterations found in GBMs.^3–5^

The epigenetic changes identified in GBMs include the silencing of genes involved in many biological processes, such as cell cycle regulation, DNA repair, and apoptosis. Among these genes are MGMT, MLH1, p16/CDKN2A, RASSF1A, PTEN, TP53, p14ARF, AR, WT1, CDH1, p15/CDKN2B, MT1A, and RB1.^6,7^ The stem cell theory of cancer proposes that cancers arise from stem cells that are present in all tissues.^8–10^

According to this theory, cancerous tissues are similar to normal tissues, being are composed of stem cells, transit-amplifying cells, and terminally differentiated cells.^11^ In recent years, various studies have reported the presence of stem cells in brain tumors, referred to as cancer stem cells (CSCs).^7–13^ CSCs are multipotent, meaning they have the property of self-renewal and are believed to be responsible for initiating and maintaining tumors, recurrence, and therapy resistance.^13,14^

The presence of stem cell-like phenotypes implies the presence of molecular networks governing them. Several recent studies have detected the expression of pluripotency-associated genes such as OCT4, SOX2, and NANOG in human tumors.^15–17^ Together, these genes are responsible for the regulation of several hundred genes involved in establishing pluripotency and stem cell differentiation *in vitro*.^18^

OCT4 and NANOG are homeobox transcription factors and SOX2 is a member of the Sox (SRY-related HMG box) gene family, which encodes transcription factors with a single HMG DNA binding domain.^19^

These three factors form a core regulatory network that coordinately determines embryonic stem cell (ESC) self-renewal and differentiation. Up or downregulation of OCT4 and NANOG induced *in vitro* may allow the entry of undifferentiated cells into differentiation pathways. In human stem cells, the reduction of OCT4 expression promotes upregulation of mesoderm and endoderm markers, whereas increased levels of OCT4 promote upregulation of endoderm markers.^20^ Another important gene recently associated with the pluripotency phenotype is the coactivator-associated arginine methyltransferase 1 – CARM1 gene, also called PRMT4.^21^

Torres-Padilla et al.^22^ demonstrated that CARM1 is required for self-renewal and pluripotency during early embryonic development. They also demonstrated that CARM1 is associated with the Oct4 and Sox2 promoters, which display detectable levels of H3 R17 and R26 methylation. During early development, Carm1 seems to exert a positive influence on the expression levels of Nanog and Sox2 genes.^22^ CARM1 is also associated with the activation function of the tumor suppressor gene TP53, acting directly with its protein sequence.^23^ CARM1 downregulation induced by RNAi causes loss of the pluripotency phenotype in cells after knockdown.^24^

These four genes are key components of the complex circuit involved in stem cells self-renewal and pluripotency, which can also act in undifferentiated tumors.^19,25^ Additional supporting evidence of the action of stem cell-related genes in tumors has been presented in many studies, which have identified a stem cell-like signature in poorly differentiated tumors, including glioblastoma. Reacquired stem cell-like characteristics can involve drug resistance, self-renewal, and embryonic-like gene expression signatures.^14^

A regulatory relationship between pluripotency cell markers and drug resistance was reported by Chambers et al.,^25^ who showed that Oct4, when overexpressed by a 3-fold factor, binds to the promoter region of ABCB1, ABCG2, and ABCC1 in MDR K562-Lucena cells. In contrast to all the undesired properties of this embryonic molecular network, some favorable factors can be present. These factors act on thousands of developmental genes, and experimental disruption of some of these factors causes differentiation of disrupted cells and loss of embryonic features.^26,27^

Understanding the common molecular networks between cancer stem cells and their normal counterparts is vital to discover the real importance of these cells in tumorigenesis processes. Advances in knowledge may help in the development of new therapeutic approaches aiming to stop the development of CSC. The aim of this study was to investigate the expression of four pluripotency-related genes (OCT4, NANOG, SOX2, and CARM1) in human glioblastoma (GBM).

## Patients and Tissue Samples

For this study, two groups of samples were investigated. One was composed glioblastoma samples from 22 patients (16 men and 6 women) who underwent tumor resection at the Clinical Hospital of Ribeirão Preto Faculty of Medicine, University of São Paulo. Tumor grade was determined according to the WHO criteria.^2,28^ The second group consisted of 10 samples of nonneoplastic white matter obtained from patients (4 men and 6 women) who underwent cortico-amygdalo-hippocampectomy for epilepsy treatment at the same hospital. The study was approved by the Ethics Committee of the Faculty of Medicine and informed consent was obtained from each patient. All tissue samples were microdissected for exclusion of tissue areas presenting necrosis or not matching GBM diagnostics prior to RNA extraction.

## Primer Design

To avoid sequence amplification of the expressed pseudogenes of NANOG and OCT4, as criticized by some authors, we performed a careful design of the primer pairs used in this study.^29,30^ The primers used for NANOG and OCT4 analysis were selected by aligning the transcribed sequences of these genes and their respective pseudogenes using the Multialin software.^31^ The primer sequences of these two genes were selected due to lack of identity with the pseudogenes and subsequent analysis with the Gene Runner v.3.05 software (Hastings Software Inc.).

The other primer sequences used were designed using GeneRunner (CARM1 gene) or selected from the work of Valente et al. (TBP and HPRT1 genes).^32^ The 5′–3′ sequences of all forward (F) and reverse (R) primers used were: OCT4A-F TCCCTTCGCAAGCCCTCAT and OCT4A-R CACCACCTGGAGGGGGCG; NANOG-F TTATAAATCTAGAGACTCCAGG and NANOG-F GAGAAATA GGACCTCCAGAAG; CARM1-F CTACCTCCACGCCAAGAAG and CARM1-R GGTGAACTGCTCCATGTAGA; HPRT1-F TGAGGATTTGGAAAGGGTGT and HPRT1-R GAG CACACAGAGGGCTACAA; TBP-F GAGCTGTGATGTG AAGTTTCC and TBP-R TCTGGGTTTGATCATTCTGTAG; β-ACTIN-F CTGCTTCCAGCTCCTCCC and β-ACTIN-R AGT TTCGTGGATGCCACAGG; GAPDH-F GTCGCCAGCCGAGC CACA and GAPDH-R GGGTGGAATCATATTGGAACA.

## Total RNA Extraction and cDNA Synthesis

Total cellular RNA was extracted using Trizol® reagent (Invitrogen) and subsequently submitted to DNase I treatment to eliminate any possible contamination with genomic DNA (gDNA). Samples of 500 ηg of total RNA were digested using 1U of DNAse I (Invitrogen) at room temperature for 15 min and inactivated by the addition of 1 µl of EDTA (25 mM) and incubated to 65°C for 5 min at a final volume of 10 µl. The DNase I treated RNA was reverse transcribed to single-stranded cDNA using a High-Capacity Kit (Applied Biosystems) according to the manufacturer's protocol.

## Qualitative and Quantitative PCR (qPCR)

The cDNA samples produced were diluted 10-fold and tested for possible gDNA contamination by amplification of a fragment of the human β-actin gene amplified with a primer pair anchored in two different exons with an intervening intron. After finding no gDNA contamination, the samples were used for real-time polymerase chain reaction (PCR) (unpublished data). The transcript levels of each studied gene were evaluated using an ABI 7500 system (Applied Biosystems).

For relative quantification (RQ) of gene expression, standard curves were constructed for each gene by considering at least 3 points in triplicate of the 10-fold serial dilution of cDNA in water, starting from a 1:10 volume of undiluted cDNA transcribed from 500 ηg of total RNA. To normalize differences in the amount of total cDNA added to each reaction, TBP and HPRT gene expression were used as endogenous control. As a calibrator sample (reference sample for relative quantification), the NTERA2 cell line was used. All reactions were performed in duplicate, and all procedures were carried out at 4°C.

## Statistics

GBMs and control samples were compared using the Mann–Whitney test, except for OCT4A analysis, in which the data were submitted to Student's t-test. All tests were two-tailed and statistical significance was considered to be *P* < .05.

## Results

### Primer Efficiency of OCT4, NANOG, SOX2, and CARM1

The primer efficiency of real-time PCR (10-1/slope−1) of the set of primer pairs used for this study ranged from 95% to 100%.

### Analysis of the Pluripotency-related Genes Expression in Glioblastomas

The quantitative results from the quantitative RT-PCR also demonstrated the presence of OCT4 transcripts in two control samples (Figure 2). The relative quantification (RQ) of OCT4 transcripts in GBM versus control samples demonstrated a 14.77-fold increase in GBMs, besides the large difference between the median of the two groups, which was statistically significant according to Student's t-test (*P* = .3561; *P* < .05). Despite no differences of expression being found, SOX2 was present in 91.66% of the analyzed samples and codetected with OCT4 in 70.83% of them (Figure 3). Analysis of the CARM1 expression comparing the RQ medians showed a 2.84-fold increase in the GBM samples versus the control. The result of the analysis of CARM1 expression demonstrated a significant difference between the groups analyzed (Mann–Whitney, test *P* = .0201; *P* < 0.05) (Figure 2). NANOG expression was not detected in either the GBMs or the control samples. The detection of NANOG sequences in tumors has been attributed to its pseudogene number 8, which is not expressed in NTERA2 cells (the calibrator sample used in this study), which were positive for NANOG expression in our assays (unpublished data).

**Figure 1.**
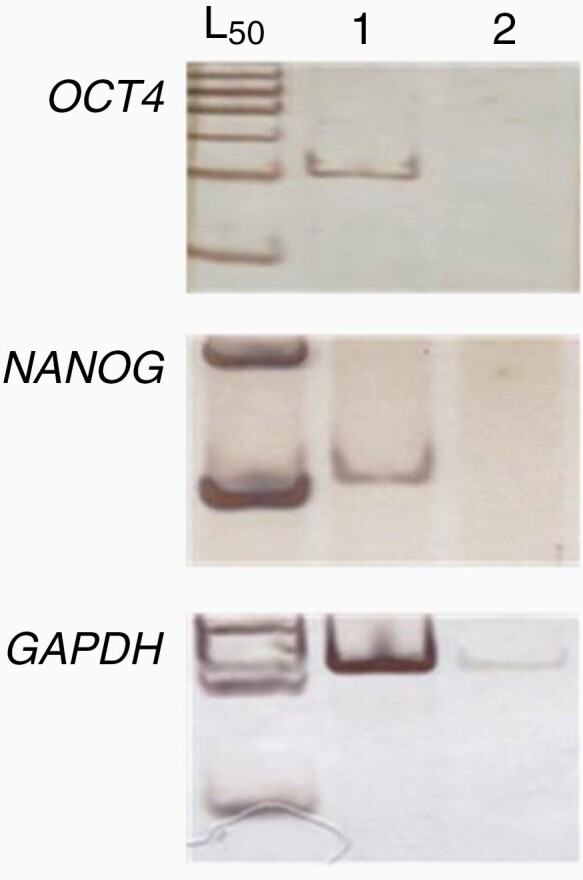
RT-PCR analysis of specific amplification by specific primer pairs of OCT4A and NANOG transcripts in: (1) NTERA2, an undifferentiated cell expressing NANOG and OCT4A but not NANOGP8; (2) peripheral blood cell cDNA (differentiated cell pool), negative for OCT4A [11, 25]. L50 – ladder 50 base pairs.

**Figure 2.**
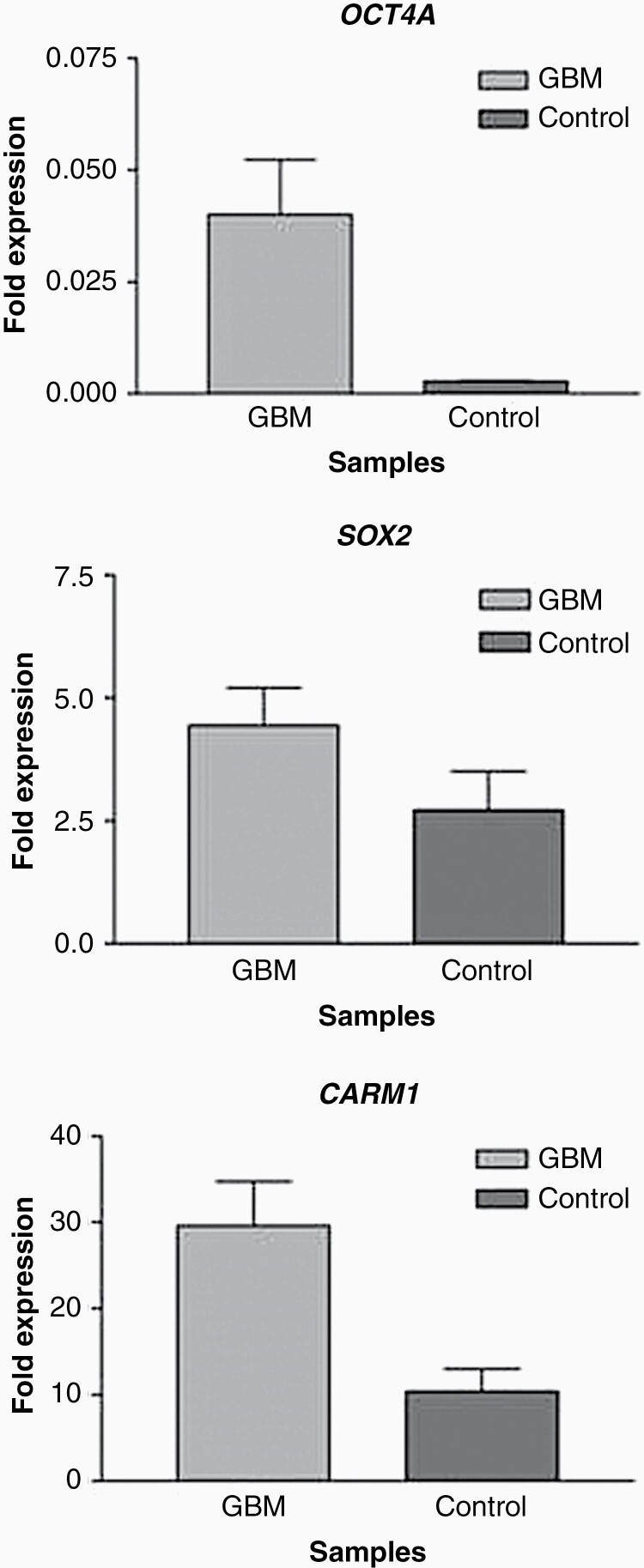
Graphic representation of differences of fold expression between GBM and control samples derived from quantitative RT-PCR of the genes: (a) OCT4 (*P* = .3561, *P* < .05); and (c) CARM1 (*P* = .0201, *P* < .05).

**Figure 3.**
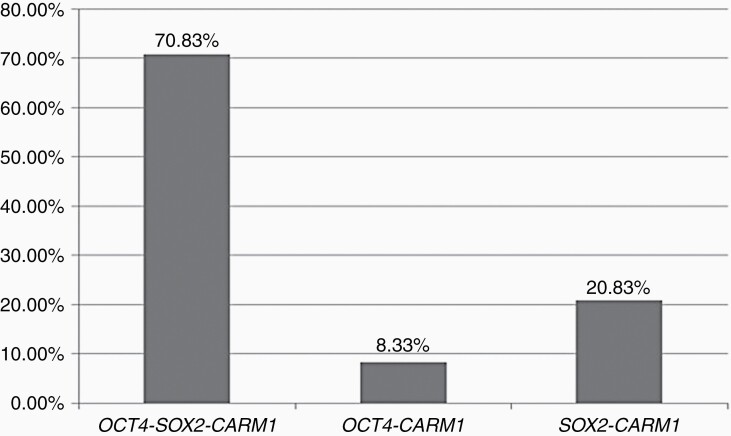
Percentage of GBM samples presenting one of the respective molecular signatures: OCT4-SOX2-CARM1 (70.83%), OCT4-CARM1(8.33%), and SOX2-CARM1 (20.83%).

## Discussion

The interpretation of the data of the expression of pluripotency-related genes in cancer has been brought into question because of the large number of processed pseudogenes or even duplicated sequences present in the case of these genes.^30,33^ Of the 22 GBM samples analyzed, 19 (86.36%) displayed OCT4 expression. Our results diverge slightly from the findings of Du et al., who found 100% (19/19) of GBM samples positive for OCT4.^34^ However, the primers used by those authors did not present a significant difference between the OCT4 and their pseudogenes (our unpublished data).

The expression of OCT4 pseudogenes has been detected in cancer cell lines as well as cancer tissues.^35,36^ An additional point that needs to be verified is the presence of two isoforms of OCT4 genes. Cauffman et al. reported in detail the two main splice variants (OCT4A and OCT4B). Functionally, the two human OCT4 isoforms showed different expression profiles, with only OCT4A being expressed in hESC.^37^

These isoforms also differ in their ability to confer self-renewal. I contrast to OCT4A, OCT4B does not have this property or capacity to bind to the OCT4 consensus binding sequence.^38^ The human OCT4A isoform is clearly located in the nucleus, whereas OCT4B is mainly expressed in the cytoplasmic compartment of the cell.^38^ The study of the specific OCT4A transcript was focused to better investigate whether a real molecular embryonic signature was present in GBMs. Together with SOX2 factors, OCT4 acts on the regulation of thousands of genes by dimer-formation and even cooccupancy of regulatory regions in the genome.^39–41^ Using data generated from ChIP-seq in GBM cells, Fang et al. found 2323 binding sites of OCT4 mapped within -2kb of the TSSs in glioblastoma cancer cells.^42^ By enrichment analysis using Gene Ontology (GO), these authors found that the genes bound by OCT4 are involved in some important biological processes, including gene expression, translation, mRNA processing, wound healing, and apoptosis.^42^ The number of genes bound by OCT4 and the biological processes associated with them provide important evidence of OCT4's role in GBM biology. In addition, as further evidence of the functional role of OCT4 in GBM, the inhibition of OCT4 and AKT potently suppresses the propagation of glioblastoma cell lines U87 and U251 *in vitro*.^43^

The coexpression of OCT4 and SOX2 in the context of *in vivo* GBM cells is also important to clarify the role of these genes in important biological processes associated with the development of these brain tumors. Both OCT4 and SOX2 expression are detected in glioblastoma stem cells (GSCs), which survive chemical treatment. These surviving GSCs have the capacity to recapitulate the tumor.^44^

SOX2 plays a critical role in the carcinogenesis and maintenance of GBM stem cells, associated with resistance to chemotherapy and radiotherapy.^45^ The same authors reported that increased SOX2 expression enhanced stem cell potency in GBM cell lines, while downregulation of this gene was associated with abrogated ability of tumor initiation and drug resistance of CD133+ GBM cells.^45^

The results of biological function and overexpression of CARM1 presented here may indicate a new therapeutic target to attack human glioblastoma.

CARM1, which methylates arginine residues at histone 3, is targeted to promoter sequences of the Oct4, Sox2, and Nanog genes, shown to be intrinsically involved in pluripotency.^24^ Downregulation of CARM1 in murine ESCs causes the cell to enter the differentiation pathways. Reduction of the transcribed level of CARM1 by RNAi, and consequently their codified protein, causes the reduction in transcriptional levels of Oct4, Sox2, and Nanog, and an increase in the expression of differentiation-associated genes.^24^

CARM1 is associated with the regulation of OCT4, SOX2, and NANOG, as indicated by the downregulation of these three genes in human embryonic stem cells submitted to knockdown of CARM1.^46^ Conversely, overexpression of CARM1 increases cells' resistance to differentiation.

Similar to our results, increased CARM1 expression was found in glioblastoma samples by Kappadakunnel et al.^47^ They reported increased expression of the CARM1 gene in GBM tumors in contact with the subventricular zone (SVZ), and associated this altered expression pattern with poorer patient survival.

Increased CARM1 expression and/or activity have previously been reported in many different cancer types, including prostate, breast, colorectal, lung, and liver cancer.^48–50^ In solid tumors, CARM1 acts as a cofactor for the transcription factor NF-κB, p53, steroid hormone receptors and, it is functionally related to cancer cell proliferation, metastasis, and poor survival outcomes.^51^

The expression of NANOG has also been detected in many cancers, but similar to OCT4, it has many pseudogenes anchored at different chromosomal positions.^46^ For the NANOG primer pair, the results also demonstrated primer specificity for amplification of this gene. In addition to the bioinformatics data (Electronic Supplementary Material Fig.1), the RT-PCR results demonstrated the amplification of the NANOG fragment from NTERA2 cDNA (Figure 1), which expresses NANOG but not NANOGP8.^16^ Early pluripotency signatures were reported by Kashyap et al. for genes regulated by OCT4, SOX2, and NANOG.^52^ In the present work, two of the three master regulators of pluripotency expressed in human glioblastoma were found, as well as increased expression of CARM1, which is involved in the regulation of both factors.^44^

Knockdown induced by shRNAs against CARM1 mRNA in glioblastoma (LN229) and neuroblastoma [BE(2)-C] cell lines inhibited the proliferation in both cell types, demonstrating the functional impact of this gene in both brain tumors.^53^ These authors also found that CARM1 downregulation was associated with decreasing mRNA or protein levels of LDHA, GLUT1, and ASCT2 genes. The results of Wang et al. suggest that CARM1 may be involved in the regulation of glycolysis and glutaminolysis in glioblastoma and neuroblastoma. Since both these metabolic pathways promote cell proliferation in multiple cancers,^54^ they could contribute to molecular mechanisms associated with the effects of CARM1 in the cell proliferation of these tumors.

The great number of genes whose expression is affected by these genes and the results of functional studies manipulating their expression suggest that these may be involved in the stem cell features present in GBMs. The biological significance of some of these factors for pluripotency can be observed from studies of the production of induced pluripotent stem (iPS) cells. The simultaneous presence of OCT4 and SOX2 was shown to be essential to achieve success in iPS cell production, and sometimes the use of only these two factors was sufficient.^19^

The central position occupied by OCT4, SOX2, and CARM1 in the control of pluripotency in undifferentiated cells may point the way to valuable opportunities to disrupt cell phenotypes associated with the survival and proliferation of cancer stem cells. Loss of these factors could disrupt important characteristics present in undifferentiated cells, such as self-renewal, drug resistance, and blocking of differentiation pathways. In this context, the expression of these three factors may indicate specific and effective targets for therapeutic intervention. Early knockdown experiments in cancer cells demonstrated that disruption of the gene expression of OCT4 caused differentiation in embryo carcinoma.^27^ Knockdown of SOX2 stopped proliferation and induced the loss of tumorigenicity in immunodeficient mice.^15^

In addition to the aforementioned data, it is important to note that many of the molecular signatures of these genes were found in studies using GBM cell lines from commercial sources or cell cultures derived from GBM tumors. In our study, we analyzed these molecular signatures in samples derived from tumor tissues obtained directly from patients, without any manipulation by *in vitro* culture protocols, reinforcing the presence of a pluripotent molecular signature in GBM cells *in vivo*. So, we believe that the same functional effects derived from the expression or the knockdown of these genes observed in *in vitro* culture studies may occur *in vivo* as well. The gene expression patterns found in our study in GBM samples indicate the occurrence of a stem cell-like molecular signature and identify these genes as possible targets in future therapeutic protocols.

## Conclusions

Embryonic stem cell gene expression signatures for OCT4A, SOX2, and CARM1 genes in GBM samples were found, and given their well-documented influences on an array of downstream targets, we suggest that the expression of these factors can be involved in the embryonic-like features present in these tumors, as previously documented by other authors.^46^ We also suggest that the expression of OCT4A, in two nonneoplasic samples, could represent a possible stem cell niche with at least one embryonic feature in adult brain tissue. As far as we know, this is the first report of the increased gene expression profile of CARM1 in addition to a stem cell-like signature involving SOX2 and OCT4 expression in GBM.

## Notes

**Authorship Statement**: Álvaro Fabrício Lopes Rios - was involved in the acquisition and analysis of the data, manuscript writing. Daniela Pretti da Cunha Tirapelli - was involved in the conception and design intellectual and scientific content, supervision of all phases of the study, and critical revision. Mucio Luiz de Assis Cirino – was involved in manuscript drafting and critical review of intellectual content. Andressa Romualdo Rodrigues – was involved in manuscript drafting and critical review of intellectual content. Ester S. Ramos - was involved in the conception and design and scientific content of the study, supervision of all phases, and critical revision. Carlos Gilberto Carlotti Jr – was involved in the conception and design and scientific content of the study, supervision of all phases, and critical revision.

## References

[1] Miller CR , Perry A. Glioblastoma. Arch Pathol Lab Med. 2007; 131(3):397–406. 17516742 10.5858/2007-131-397-G

[2] Louis DN , Perry A, Reifenberger G, et al. The 2016 World Health Organization Classification of tumors of the central nervous system: a summary. Acta Neuropathol. 2016; 131(6):803–820. 27157931 10.1007/s00401-016-1545-1

[3] Kanu OO , Hughes B, Di C, et al. glioblastoma multiforme oncogenomics and signaling pathways. Clin Med Oncol. 2009; 3:39–52. 19777070 10.4137/cmo.s1008 PMC2748278

[4] Kalkan R . The importance of mutational drivers in GBM. Crit Rev Eukaryot Gene Expr. 2016; 26(1):19–26. 27278882 10.1615/CritRevEukaryotGeneExpr.v26.i1.30

[5] Wang J , Su HK, Zhao HF, Chen ZP, To SS. Progress in the application of molecular biomarkers in gliomas. Biochem Biophys Res Commun. 2015; 465(1):1–4. 26253473 10.1016/j.bbrc.2015.07.148

[6] Burgess R , Jenkins R, Zhang Z. Epigenetic changes in gliomas. Cancer Biol Ther. 2008; 7(9):1326–1334. 18836290 10.4161/cbt.7.9.6992 PMC2954629

[7] Xu Q , Yuan X, Tunici P, et al. Isolation of tumour stem-like cells from benign tumours. Br J Cancer. 2009; 101(2):303–311. 19568241 10.1038/sj.bjc.6605142 PMC2720199

[8] Yi L , Zhou ZH, Ping YF, et al. Isolation and characterization of stem cell-like precursor cells from primary human anaplastic oligoastrocytoma. Mod Pathol. 2007; 20(10):1061–1068. 17660801 10.1038/modpathol.3800942

[9] Tuncel G , Kalkan R. Receptor tyrosine kinase-Ras-PI 3 kinase-Akt signaling network in glioblastoma multiforme. Med Oncol. 2018; 35(9):122. 30078108 10.1007/s12032-018-1185-5

[10] Yuan X , Curtin J, Xiong Y, et al. Isolation of cancer stem cells from adult glioblastoma multiforme. Oncogene. 2004; 23(58):9392–9400. 15558011 10.1038/sj.onc.1208311

[11] Sell S . On the stem cell origin of cancer. Am J Pathol. 2010; 176(6):2584–2494. 20431026 10.2353/ajpath.2010.091064 PMC2877820

[12] Singh SK , Clarke ID, Terasaki M, et al. Identification of a cancer stem cell in human brain tumors. Cancer Res. 2003; 63(18):5821–5828. 14522905

[13] Bao S , Wu Q, McLendon RE, et al. Glioma stem cells promote radioresistance by preferential activation of the DNA damage response. Nature. 2006; 444(7120):756–760. 17051156 10.1038/nature05236

[14] Liu G , Yuan X, Zeng Z, et al. Analysis of gene expression and chemoresistance of CD133+ cancer stem cells in glioblastoma. Mol Cancer. 2006; 5:67. 17140455 10.1186/1476-4598-5-67 PMC1697823

[15] Gangemi RM , Griffero F, Marubbi D, et al. SOX2 silencing in glioblastoma tumor-initiating cells causes stop of proliferation and loss of tumorigenicity. Stem Cells. 2009; 27(1):40–48. 18948646 10.1634/stemcells.2008-0493

[16] Jeter CR , Badeaux M, Choy G, et al. Functional evidence that the self-renewal gene NANOG regulates human tumor development. Stem Cells. 2009; 27(5):993–1005. 19415763 10.1002/stem.29 PMC3327393

[17] Schoenhals M , Kassambara A, De Vos J, Hose D, Moreaux J, Klein B. Embryonic stem cell markers expression in cancers. Biochem Biophys Res Commun. 2009; 383(2):157–162. 19268426 10.1016/j.bbrc.2009.02.156

[18] Boyer LA , Lee TI, Cole MF, et al. Core transcriptional regulatory circuitry in human embryonic stem cells. Cell. 2005; 122(6):947–956. 16153702 10.1016/j.cell.2005.08.020 PMC3006442

[19] Loh YH , Ng JH, Ng HH. Molecular framework underlying pluripotency. Cell Cycle. 2008; 7(7):885–891. 18414030 10.4161/cc.7.7.5636

[20] Rodriguez RT , Velkey JM, Lutzko C, et al. Manipulation of OCT4 levels in human embryonic stem cells results in induction of differential cell types. Exp Biol Med (Maywood). 2007; 232(10):1368–1380. 17959850 10.3181/0703-RM-63

[21] Bauer UM , Daujat S, Nielsen SJ, Nightingale K, Kouzarides T. Methylation at arginine 17 of histone H3 is linked to gene activation. EMBO Rep. 2002; 3(1):39–44. 11751582 10.1093/embo-reports/kvf013 PMC1083932

[22] Torres-Padilla ME , Parfitt DE, Kouzarides T, Zernicka-Goetz M. Histone arginine methylation regulates pluripotency in the early mouse embryo. Nature. 2007; 445(7124):214–218. 17215844 10.1038/nature05458 PMC3353120

[23] An W , Kim J, Roeder RG. Ordered cooperative functions of PRMT1, p300, and CARM1 in transcriptional activation by p53. Cell. 2004; 117(6):735–748. 15186775 10.1016/j.cell.2004.05.009

[24] Wu Q , Bruce AW, Jedrusik A, et al. CARM1 is required in embryonic stem cells to maintain pluripotency and resist differentiation. Stem Cells. 2009; 27(11):2637–2645. 19544422 10.1002/stem.131 PMC4135545

[25] Chambers I , Smith A. Self-renewal of teratocarcinoma and embryonic stem cells. Oncogene. 2004; 23(43):7150–7160. 15378075 10.1038/sj.onc.1207930

[26] Chambers I , Colby D, Robertson M, et al. Functional expression cloning of Nanog, a pluripotency sustaining factor in embryonic stem cells. Cell. 2003; 113(5):643–655. 12787505 10.1016/s0092-8674(03)00392-1

[27] Matin MM , Walsh JR, Gokhale PJ, et al. Specific knockdown of Oct4 and beta2-microglobulin expression by RNA interference in human embryonic stem cells and embryonic carcinoma cells. Stem Cells. 2004; 22(5):659–668. 15342930 10.1634/stemcells.22-5-659

[28] Louis DN , Ohgaki H, Wiestler OD, et al. The 2007 WHO classification of tumours of the central nervous system. Acta Neuropathol. 2007; 114(2):97–109. 17618441 10.1007/s00401-007-0243-4 PMC1929165

[29] Liedtke S , Enczmann J, Waclawczyk S, Wernet P, Kögler G. Oct4 and its pseudogenes confuse stem cell research. Cell Stem Cell. 2007; 1(4):364–366. 18371374 10.1016/j.stem.2007.09.003

[30] Liedtke S , Stephan M, Kögler G. Oct4 expression revisited: potential pitfalls for data misinterpretation in stem cell research. Biol Chem. 2008; 389(7):845–850. 18627312 10.1515/BC.2008.098

[31] Corpet F . Multiple sequence alignment with hierarchical clustering. Nucleic Acids Res. 1988; 16(22):10881–10890. 2849754 10.1093/nar/16.22.10881 PMC338945

[32] Valente V , Teixeira SA, Neder L, et al. Selection of suitable housekeeping genes for expression analysis in glioblastoma using quantitative RT-PCR. BMC Mol Biol. 2009; 10:17. 19257903 10.1186/1471-2199-10-17 PMC2661085

[33] Sneha S , Nagare RP, Manasa P, Vasudevan S, Shabna A, Ganesan TS. Analysis of human stem cell transcription factors. Cell Reprogram. 2019; 21(4):171–180. 31298562 10.1089/cell.2019.0005

[34] Du Z , Jia D, Liu S, et al. Oct4 is expressed in human gliomas and promotes colony formation in glioma cells. Glia 2009; 57(7):724–733. 18985733 10.1002/glia.20800

[35] Suo G , Han J, Wang X, et al. Oct4 pseudogenes are transcribed in cancers. Biochem Biophys Res Commun. 2005; 337(4):1047–1051. 16229821 10.1016/j.bbrc.2005.09.157

[36] van Schaijik B , Davis PF, Wickremesekera AC, Tan ST, Itinteang T. Subcellular localisation of the stem cell markers OCT4, SOX2, NANOG, KLF4 and c-MYC in cancer: a review. J Clin Pathol. 2018; 71(1):88–91. 29180509 10.1136/jclinpath-2017-204815

[37] Cauffman G , Liebaers I, Van Steirteghem A, Van de Velde H. POU5F1 isoforms show different expression patterns in human embryonic stem cells and preimplantation embryos. Stem Cells. 2006; 24(12):2685–2691. 16916925 10.1634/stemcells.2005-0611

[38] Lee J , Kim HK, Rho JY, Han YM, Kim J. The human OCT-4 isoforms differ in their ability to confer self-renewal. J Biol Chem. 2006; 281(44):33554–33565. 16951404 10.1074/jbc.M603937200

[39] Botquin V , Hess H, Fuhrmann G, et al. New POU dimer configuration mediates antagonistic control of an osteopontin preimplantation enhancer by Oct-4 and Sox-2. Genes Dev. 1998; 12(13):2073–2090. 9649510 10.1101/gad.12.13.2073 PMC316977

[40] Nishimoto M , Fukushima A, Okuda A, Muramatsu M. The gene for the embryonic stem cell coactivator UTF1 carries a regulatory element which selectively interacts with a complex composed of Oct-3/4 and Sox-2. Mol Cell Biol. 1999; 19(8):5453–5465. 10409735 10.1128/mcb.19.8.5453 PMC84387

[41] Rodda DJ , Chew JL, Lim LH, et al. Transcriptional regulation of nanog by OCT4 and SOX2. J Biol Chem. 2005; 280(26):24731–24737. 15860457 10.1074/jbc.M502573200

[42] Fang XF , Zhang WY, Zhao N, et al. Genome-wide analysis of OCT4 binding sites in glioblastoma cancer cells. J Zhejiang Univ Sci B. 2011; 12(10):812–819. 21960344 10.1631/jzus.B1100059 PMC3190096

[43] Li W , Zhou Y, Zhang X, et al. Dual inhibiting OCT4 and AKT potently suppresses the propagation of human cancer cells. Sci Rep. 2017; 7:46246. 28383051 10.1038/srep46246 PMC5382782

[44] Guerra-Rebollo M , Garrido C, Sánchez-Cid L, et al. Targeting of replicating CD133 and OCT4/SOX2 expressing glioma stem cells selects a cell population that reinitiates tumors upon release of therapeutic pressure. Sci Rep. 2019; 9(1):9549. 31267022 10.1038/s41598-019-46014-0 PMC6606606

[45] Song WS , Yang YP, Huang CS, et al. Sox2, a stemness gene, regulates tumor-initiating and drug-resistant properties in CD133-positive glioblastoma stem cells. J Chin Med Assoc. 2016; 79(10):538–545. 27530866 10.1016/j.jcma.2016.03.010

[46] Xu Z , Jiang J, Xu C, et al. MicroRNA-181 regulates CARM1 and histone arginine methylation to promote differentiation of human embryonic stem cells. PLoS One. 2013; 8(1):e53146. 23301034 10.1371/journal.pone.0053146 PMC3536801

[47] Kappadakunnel M , Eskin A, Dong J, et al. Stem cell associated gene expression in glioblastoma multiforme: relationship to survival and the subventricular zone. J Neurooncol. 2010; 96(3):359–367. 19655089 10.1007/s11060-009-9983-4 PMC2808508

[48] Kim YR , Lee BK, Park RY, et al. Differential CARM1 expression in prostate and colorectal cancers. BMC Cancer. 2010; 10:197. 20462455 10.1186/1471-2407-10-197 PMC2881889

[49] Al-Dhaheri M , Wu J, Skliris GP, et al. CARM1 is an important determinant of ERα-dependent breast cancer cell differentiation and proliferation in breast cancer cells. Cancer Res. 2011; 71(6):2118–2128. 21282336 10.1158/0008-5472.CAN-10-2426 PMC3076802

[50] Osada S , Suzuki S, Yoshimi C, et al. Elevated expression of coactivator-associated arginine methyltransferase 1 is associated with early hepatocarcinogenesis. Oncol Rep. 2013; 30(4):1669–1674. 23912631 10.3892/or.2013.2651

[51] Mann M , Cortez V, Vadlamudi R. PELP1 oncogenic functions involve CARM1 regulation. Carcinogenesis. 2013; 34(7):1468–1475. 23486015 10.1093/carcin/bgt091 PMC3697892

[52] Kashyap V , Rezende N, Scotland K, et al. Regulation of stem cell pluripotency and differentiation involves a mutual regulatory circuit of the Nanog, OCT4, and SOX2 Pluripotency transcription factors with polycomb repressive complexes and stem cell microRNAs. Stem Cell Dev. 2009; 18(7):1093–1108. 10.1089/scd.2009.0113 PMC3135180 19480567

[53] Wang F , Zhang J, Ke X, et al. WDR5-Myc axis promotes the progression of glioblastoma and neuroblastoma by transcriptional activating CARM1. Biochem Biophys Res Commun. 2020; 523(3):699–706. 31948749 10.1016/j.bbrc.2019.12.101

[54] Stine ZE , Walton ZE, Altman BJ, Hsieh AL, Dang CV. MYC, metabolism, and cancer. Cancer Discov. 2015; 5(10):1024–1039. 26382145 10.1158/2159-8290.CD-15-0507 PMC4592441

